# Selecting the most suitable organizational structure for hospitals: an integrated fuzzy FUCOM-MARCOS method

**DOI:** 10.1186/s12962-022-00362-3

**Published:** 2022-06-27

**Authors:** Mohsen Khosravi, Arash Haqbin, Zahra Zare, Payam Shojaei

**Affiliations:** 1 Department of Health Services Management, Shiraz University of Medical Sciences, Shiraz, Iran; 2 Department of Management, Shiraz University, Shiraz, Iran

**Keywords:** Organizational structure, Structural adjustment policy, Hospitals, Fuzzy set theory, FUCOM, MARCOS

## Abstract

**Background:**

Previous studies mentioned four organizational structures for hospitals, which are budgetary, autonomous, corporate, and private. Nevertheless, healthcare decision-makers are still required to select the most organizational structure specific to their circumstances. The present study aims to provide a framework to prioritize and select the most suitable organizational structure using multicriteria decision-making (MCDM) methods in Iranian hospitals.

**Methods:**

First, a multicriteria decision-making model consisted of the respective criteria, and alternatives were developed. The pertinent criteria were identified through a systematic literature review. The coefficient weights of the identified criteria were then calculated using FUCOM-F. Finally, organizational structures were prioritized in accordance with the identified criteria using FMARCOS.

**Results:**

The findings reveal that income is the most significant criterion in selecting organizational structures for hospitals whereas the number of outpatient visits is the least important. Also, the private structure is the most appropriate, and budgetary style is the least suitable organizational structure for Iranian hospitals.

**Conclusion:**

Providing a framework in order to select the most appropriate organizational structure could help managers and policymakers of the healthcare sector in Iran and other countries, mainly similar developing countries.

## Introduction

Throughout history, numerous organizational modalities have been introduced worldwide. These organizational modalities and the efforts made in order to refine them brought about various extensive changes, from a simple transition to a national revolution, which shows the importance of structure in organizations and institutions [1]. Nowadays, despite the approximate integration of these modalities after World War II (In particular, after the collapse of the Eastern Bloc), the debate over these structural changes is being continued. Such reforms are still being proposed to different countries, especially to developing countries with low income, under various titles such as structural adjustment reforms by international institutions like World Bank and International Monetary Fund. Naturally, the healthcare sector of the countries is also affected by these reforms, and, as a result, the organizational modality of health care providers changes [2].

In a study conducted by the World Bank [3], four types of hospitals in terms of the organizational modality were enumerated, which includes budgetary hospitals (with the lowest independence and minimal market connection), autonomous hospitals, corporate hospitals, and private hospitals (with the most independence from the government and the most connection with the market). According to the mentioned study, autonomization, corporatization, and privatization have the elements of marketization, which means reducing government direct control over hospitals and increasing their links to the market or market-like incentives. On the other hand, under the budgetary style, hospitals are often run as part of the government and have a designated and centralized budget, and all revenue goes back to a central ministry. Managers in budgetary hospitals have a degree of control. In contrast, autonomous public hospitals focus on marketing management, giving managers varying degrees of control over most daily decisions, increasing the organization's share of revenue, and exposing the organization to some degree of market or market-like pressures. Moreover, corporatization takes organizational reform one step further by mimicking the structure and efficiency of private companies, giving managers more control over decisions, service delivery, net income, and exposing the hospital to the market while emphasizing social goals through public ownership. Finally, privatization turns the public hospital into a for-profit or a non-profit private hospital. Full interaction with the market for revenue and high motivation of the owner to earn has increased the popularity of privatization compared to other structural styles [4].

Researchers and practitioners that prescribe structural adjustment policies for organizations and institutions believe that applying such structural adjustments in organizations will lead to organizational development, performance improvement, and ease of achieving organizational goals in the organization [5]. However, the debatable point in this regard is the type of structural adjustment policy applied in organizations by managers, each of which is selected according to different factors such as the environmental conditions, organizational settings according to Fiedler's contingency theory, short-term and long-term goals of the organization, and other specific circumstances [6, 7].

The existing literature on the topic assert a conflicting and sometimes controversial results regarding the issue of restructuring healthcare organizations, specially hospitals. Regarding the autonomous structure of healthcare organziations, Govindaraj and Chawla [8] conducted a study in 1996 in five countries(Ghana, Kenya, Zimbawe, India and Indonesia)on the possible effects of restructuring healthcare facilities through autonomization; Few hospitals were monitored in each of the countries and the final conclusion was that autonomization hasn’t had any effect on efficiency, quality and accountibility. Harding and Preker  [3] assessed eight countries including Brittain, New Zealand, Australia, Hong Kong, Malaysia, Singapore, Indonesia and Tunisia; their findings disclosed that due to lack of data and effects of the correlative variables, no conclusive remark can be achieved from the study. Furthermore, McPake et al. [9] monitored some positive effects of autonomization on efficiency in five hospitals in Bogota, Columbia; Even though they asserted some positive effects, they mentioned that such improvements could be as the result of payment reforms that had been occurred during the time of autonomization. Kalhori [10] conducted a study on autonomized hospitals in Iran. The results showed that bed occupancy rate and the operation ratio was below the standards and in terms of financial figures, the hospitals were bankrupt. On the issue of corporatization and the corporate structure of hospitals, Gathorn [11] conducted a survey on nurses of corporatized hospitals in the US and asserted that corporatization had negative effects on nurses behavior and some of nurses had even started to do violent behaviors towards patients after the corporatization. Moreover, Collyer et al. [12] conducted a study in Australia and asserted that there is no proof showing that corporatized hospitals and corporate structure are more efficient than public hospitals and declared that any decrease witnessed in costs after corporatization is due to the elimination of highly skilled workers and employees. Kahancova et al. [13] conducted a study in Slovakia and Hungary and declared that working situation has become worse after corporatization due to the increasing target of decreasing costs initiated by managers after restructuring the organizations; they asserted that corporatization has been used as a mean towards a fully privatized hospitals. On the issue of the possible effects of privatization and restructuring of hospitals into fully private entities, Villa and Kane [14] conducted a study to assess the effects of privatization of hospitals in three states in the US; They found that with privatization, the hospitals decreased the amount of service delivery due to less profitability of some services and therefore privatization has decreased the access to the needed services for patients. Finally, Albreht [15] conducted a study on the privatization process in Europe and concluded that there is a risk of limited access and decreased equity after implementing privatization and it may create a parallel healthcare system in which only patients with higher payments can benefit from the services.

Since there are different factors that affect the process of selecting the type of organizational modality, this problem can be looked at from multicriteria decision-making (MCDM) point of view. MCDM problems have several alternatives and the decision-maker (DM) prioritizes the alternatives based on a set of relevant criteria [16]. Accordingly, the present study aims to select the best organizational modality for public hospitals in Iran using two novel MCDM techniques in a fuzzy environment, namely Full Consistency method (FUCOM) and Measurement Alternatives and Ranking according to the Compromise Solution (MARCOS). As a powerful and novel techniques, FUCOM and MARCOS methods are used and provided successful results in various contexts such as construction project management [17], road safety assessment [18], service quality assessment [19], alternative fuel evaluation [20], human resource management [21], sustainable traffic management [22], location selection [23], project management [24], measuring supply chain performance [25], and supplier selection [26]. Moreover, the fuzziness help overcome the ambiguity and uncertainty resulting from the judgments of the experts [27].

Despite lack of evidence on the usage of MCDM techniques to probe organizational structures and their effects in the healthcare sector, there is a growing trend in usage of MCDM techniques in other areas of healthcare which motivated the authors of current study to use MCDM techniques on the current topic and paving the way for future studies to get conducted by using such techniques by filling the existing void in the literature; Ahmadi et al. [28] conducted a research to identify factors that affect the hospital decision in adopting Hospital Information System (HIS) through using a hybrid MCDM technique. Similarly, Si et al. [29] used a hybrid MCDM technique to identify Key Performance Indicators for Holistic Hospital Management in hospitals. Torkzad and Beheshtinia [30] used four hybrid methods to evaluate and prioritize hospital service quality in a sample of 4 public hospitals in Iran. Moreover, Kadoic et al. [31] measured quality of public hospitals in Croatia Using a Multi-Criteria Approach with an aim to develop a methodology for ranking top-performing hospitals at the national level.

As mentioned above, he novelty of this paper can be traced back to the MCDM methodology applied in the research, giving in a new method and platform for researchers, healthcare managers and politicians in this scope of science to probe existing options on the table regarding the issue of increasing organizational efficiency and effectiveness through restructuring healthcare organizations in a more comprehensive and evidence-based framework.

The author’s motivation for conducting such study with such methodology was to investigate the most important organizational factors in the healthcare sector and suitable organizational structures to be applied in hospitals in Iran by consideration of its unique geopolitical, ecological and socioeconomic status by using local experts living in the country beside giving in a novel comprehensive evidence-based framework for upcoming researchers in the international stage in the scope of study.

The contribution of the present study is twofold: (a) it adopts a novel methodology based on FUCOM and MARCOS in a fuzzy environment, which, to the best of the authors’ knowledge, has not been employed in the healthcare management context and can be a pioneer of the future research on such topic through using a more defined and comprehensive numerical methodology such as the abovementioned methods; and (b) it tries to provide empirical findings that could help hospital managers and healthcare policymakers to select the most appropriate hospital organizational structure in Iran in a more detailed and comprehensive way in comparison to the previous studies. The results of this study can also be generalized to other countries, mainly similar developing countries.

The remainder of this study is structured as follows. "Methods" section introduces the proposed methodology in detail. Then, in "Results" the findings are provided and the results are discussed. Finally, "Conclusion" section draws the conclusion.

## Methods

**Fig. 1:**
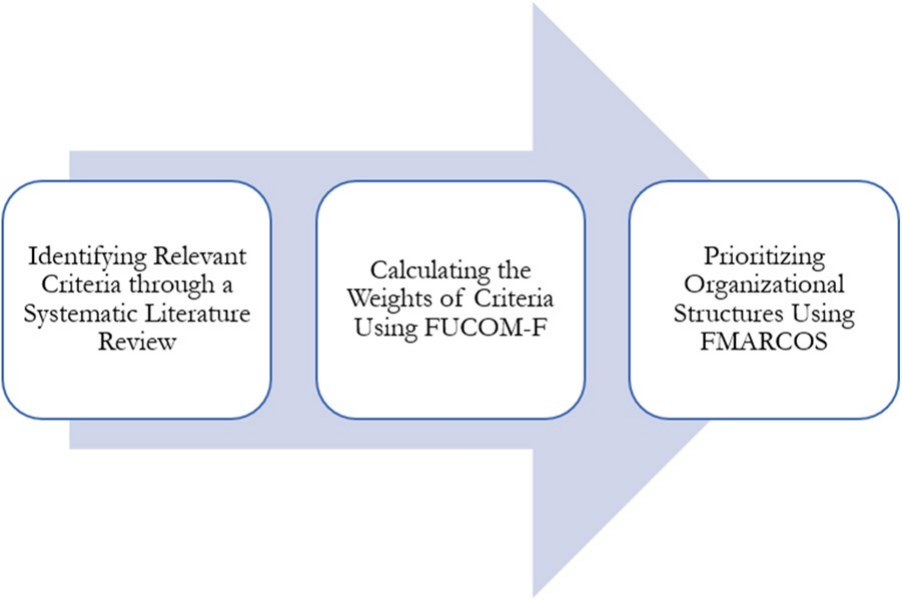
Research process

As previously mentioned, the primary purpose of this study is to choose the best organizational modality for Iranian hospitals. to achieve the mentioned purpose, the present study has consisted of three main stages, which are denoted in Fig. 1.

As illustrated in Fig. 1, first, a systematic literature review was conducted using major scientific databases such as Web of Science, Scopus, and PubMed in order to identify the affecting factors in selecting the organizational modalities for hospitals. The systematic review resulted in developing a multicriteria decision-making model consisting of decision criteria and alternatives. Then, the weights of the identified factors were calculated using Fuzzy Full Consistency (FUCOM-F) Method. Finally, the four organizational modalities mentioned earlier, namely, budgetary, autonomous, corporate, and private were prioritized in accordance with the identified criteria using Fuzzy Measurement Alternatives and Ranking according to the Compromise Solution Method (FMARCOS). Noteworthy to mention that the required data for calculating the weights and prioritizing the alternatives were gathered by pairwise comparison questionnaires from the members of an expert panel. Table 1 shows more information regarding the expert panel.

**Table 1:** Expert panel details

Expert	Expertise	Education	Experience
E1	Hospital Manager	Medical Doctor	10 years
E2	Hospital Manager	Medical Doctor	8 years
E3	Hospital Manager	Medical Doctor	7 years
E4	Professor	Ph.D. in Healthcare Management	9 years
E5	Professor	Ph.D. in Healthcare Management	5 years
E6	Researcher	Ph.D. in Healthcare Management	3 years

In the following, fuzzy set theory and each adopted technique, FUCOM-F, and FMARCOS, are further elaborated:

### Fuzzy set theory

First introduced by Zadeh in the 1960s, fuzzy set theory is an extension to classic set theory. fuzzy set theory is a membership function that plots elements to degrees of membership within a specific interval (Commonly [0, 1]). Fuzzy set theory can be extremely practical in uncertain decision-making environments and can eliminate the vagueness, ambiguity, and subjectiveness of the decision-makers (DMs), with the following main definitions [27]:

#### **Definition 1**

Assume that $\stackrel{\sim }{\upomega }\in \mathrm{F}(\mathrm{R})$ is a fuzzy number if two conditions are met. First, there is ${\mathrm{x}}_{0}\in \mathrm{R}$ such that${\upmu }_{\stackrel{\sim }{\upomega }} \left({\mathrm{x}}_{0}\right)=1$. Second, for any $\mathrm{\alpha }\in \left[0, 1\right],$ ${\stackrel{\sim }{\upomega }}_{\mathrm{\alpha }}=[\mathrm{x}, {\upmu }_{{\stackrel{\sim }{\upomega }}_{\mathrm{\alpha }}}(\mathrm{x})\ge \mathrm{\alpha }]$ is a closed interval. It should be noted that R is the set of real numbers and F(R) shows the fuzzy set.

#### **Definition 2**

A fuzzy number $\stackrel{\sim }{\upomega }$ on R is a triangular fuzzy number (TFN) if its member function ${\upmu }_{{\stackrel{\sim }{\upomega }}_{\mathrm{\alpha }}}\left(\mathrm{x}\right):\mathrm{R}\to [0, 1]$ is:

1 $${\upmu }_{{\stackrel{\sim }{\upomega }}_{\mathrm{\alpha }}}\left(\mathrm{x}\right)= \left\{\begin{array}{l}\begin{array}{ll}0, & \mathrm{ x}<\mathrm{l}\end{array}\\ \begin{array}{l}\begin{array}{ll}\frac{\mathrm{x}-\mathrm{l}}{\mathrm{m}-\mathrm{l}},& \mathrm{l}\le \mathrm{x}<\mathrm{m}\end{array}\\ \begin{array}{ll}\frac{\mathrm{u}-\mathrm{x}}{\mathrm{u}-\mathrm{m}},& \mathrm{m}\le \mathrm{x}\le \mathrm{u}\end{array}\end{array}\\ \begin{array}{ll}0,& \mathrm{ x}>\mathrm{u}\end{array}\end{array}\right.$$

where l, m, and u denote the lower, modal, and upper value of the $\stackrel{\sim }{\upomega }$ in crisp form, respectively.

#### **Definition 3**

The graded mean integration representation (GMIR) of a TFN $\stackrel{\sim }{\upomega }$ shows the ranking of that triangular fuzzy number and can be computed as:

2 $$\mathrm{R}\left({\stackrel{\sim }{\upomega }}_{\mathrm{i}}\right)= \frac{{\mathrm{l}}_{\mathrm{i}}+ {4\mathrm{m}}_{\mathrm{i}}+{\mathrm{u}}_{\mathrm{i}}}{6}$$

#### **Definition 4**

If $\stackrel{\sim }{\mathrm{A}}=({\mathrm{l}}_{\mathrm{A}},{\mathrm{m}}_{\mathrm{A}},{\mathrm{u}}_{\mathrm{A}})$ and $\stackrel{\sim }{\mathrm{B}}=\left({\mathrm{l}}_{\mathrm{B}},{\mathrm{m}}_{\mathrm{B}},{\mathrm{u}}_{\mathrm{B}}\right)$ are to TFNs, the basic mathematical operations between these two TFNs are as follows [32]:

**Addition:**

3 $$\stackrel{\sim }{\mathrm{A}}\oplus \stackrel{\sim }{\mathrm{B}}=\left({\mathrm{l}}_{\mathrm{A}},{\mathrm{m}}_{\mathrm{A}},{\mathrm{u}}_{\mathrm{A}}\right)+\left({\mathrm{l}}_{\mathrm{B}},{\mathrm{m}}_{\mathrm{B}},{\mathrm{u}}_{\mathrm{B}}\right)=({\mathrm{l}}_{\mathrm{A}}+{\mathrm{l}}_{\mathrm{B}},{\mathrm{m}}_{\mathrm{A}}+{\mathrm{m}}_{\mathrm{B}},{\mathrm{u}}_{\mathrm{A}}+{\mathrm{u}}_{\mathrm{B}})$$

**Subtraction:**

4 $$\stackrel{\sim }{\mathrm{A}}-\stackrel{\sim }{\mathrm{B}}=\left({\mathrm{l}}_{\mathrm{A}},{\mathrm{m}}_{\mathrm{A}},{\mathrm{u}}_{\mathrm{A}}\right)-\left({\mathrm{l}}_{\mathrm{B}},{\mathrm{m}}_{\mathrm{B}},{\mathrm{u}}_{\mathrm{B}}\right)=({\mathrm{l}}_{\mathrm{A}}-{\mathrm{l}}_{\mathrm{B}},{\mathrm{m}}_{\mathrm{A}}-{\mathrm{m}}_{\mathrm{B}},{\mathrm{u}}_{\mathrm{A}}-{\mathrm{u}}_{\mathrm{B}})$$

**Multiplication:**

5 $$\stackrel{\sim }{\mathrm{A}}\otimes \stackrel{\sim }{\mathrm{B}}=\left({\mathrm{l}}_{\mathrm{A}},{\mathrm{m}}_{\mathrm{A}},{\mathrm{u}}_{\mathrm{A}}\right)\otimes \left({\mathrm{l}}_{\mathrm{B}},{\mathrm{m}}_{\mathrm{B}},{\mathrm{u}}_{\mathrm{B}}\right)=({\mathrm{l}}_{\mathrm{A}}\times {\mathrm{l}}_{\mathrm{B}},{\mathrm{m}}_{\mathrm{A}}\times {\mathrm{m}}_{\mathrm{B}},{\mathrm{u}}_{\mathrm{A}}\times {\mathrm{u}}_{\mathrm{B}})$$

**Division:**

6 $$\frac{\stackrel{\sim }{\mathrm{A}}}{\stackrel{\sim }{\mathrm{B}}}= \frac{({\mathrm{l}}_{\mathrm{A}},{\mathrm{m}}_{\mathrm{A}},{\mathrm{u}}_{\mathrm{A}})}{({\mathrm{l}}_{\mathrm{B}},{\mathrm{m}}_{\mathrm{B}},{\mathrm{u}}_{\mathrm{B}})}=\left(\frac{{\mathrm{l}}_{\mathrm{A}}}{{\mathrm{u}}_{\mathrm{B}}} ,\frac{{\mathrm{m}}_{\mathrm{A}}}{{\mathrm{m}}_{\mathrm{B}}} ,\frac{{\mathrm{u}}_{\mathrm{A}}}{{\mathrm{l}}_{\mathrm{B}}}\right)$$

**Reciprocal:**

7 $${\stackrel{\sim }{\mathrm{A}}}^{-1}={({\mathrm{l}}_{\mathrm{A}},{\mathrm{m}}_{\mathrm{A}},{\mathrm{u}}_{\mathrm{A}})}^{-1}=\left(\frac{1}{{\mathrm{l}}_{\mathrm{A}}} ,\frac{1}{{\mathrm{m}}_{\mathrm{A}}} ,\frac{1}{{\mathrm{u}}_{\mathrm{A}}}\right)$$

### Fuzzy Full Consistency Method (FUCOM-F)

Full Consistency Method (FUCOM) is developed by Pamučar et al. [33], which benefits from less pairwise comparisons than other weight calculation methods such as Best–Worst Method (BWM) and Analytical Hierarchy Process (AHP) [34, 35]. The accuracy of methods for determining the weight coefficients is extremely dependent on the number of pairwise comparisons [36]. If n represents the number of criteria, then, the required pairwise comparisons for AHP and BWM are "$\frac{n(n-1)}{2}"$ and "2n−3", respectively. The number of pairwise comparisons for FUCOM, however, is only "n−1". Consequently, FUCOM must have more accurate and reliable results, which is also proved by other studies [37]. Pamučar and Ecer combined the Full Consistency Method with fuzzy set theory to develop FUCOM-F [36]. This recently developed technique is used in several contexts such as transportation management and healthcare management [18, 20, 38, 39]. In the following, steps of FUCOM-F are explained:

**Step 1.** First, a set of decision criteria will be identified, which are represented by {C1, C2, …, Cn}. Then, the decision-maker (DM) arranges the identified criteria based on their significance in a way that the first criterion is expected to be the most important whereas the last criterion is expected to be the least important.

8 $${\mathrm{C}}_{1}\ge {\mathrm{C}}_{2}\ge \cdots \ge {\mathrm{C}}_{\mathrm{n}}$$

**Step 2.** Afterward, a pairwise comparison will be done. All the criteria are mutually compared to the most significant criteria using a fuzzy linguistic scale provided in Table 2 to obtain the fuzzy criterion significance (${\stackrel{\sim }{\upomega }}_{{\mathrm{C}}_{\mathrm{n}}}$). Also, because the first-ranked criterion is compared with itself its membership function is (1, 1, 1). Using the fuzzy criterion significance (${\stackrel{\sim }{\upomega }}_{{\mathrm{C}}_{\mathrm{n}}}$), fuzzy comparative significance (${\mathrm{\varphi }}_{\mathrm{k}/(\mathrm{k}+1)}$) is computed as follows:

**Table 2:** Fuzzy linguistic terms for decision-makers (Source: [36])

Linguistic terms	Membership function
Equally Important (EI)	(1,1, 1)
Weakly Important (WI)	(2/3, 1, 3/2)
Fairly Important (FI)	(2/5, 2, 2/3)
Very Important (VI)	(2/7, 3, 2/5)
Absolutely Important (AI)	(2/9, 4, 2/7)

9 $${\mathrm{\varphi }}_{\mathrm{k}/(\mathrm{k}+1)}=\frac{{\stackrel{\sim }{\upomega }}_{{\mathrm{C}}_{(\mathrm{k}+1)}}}{{\stackrel{\sim }{\upomega }}_{{\mathrm{C}}_{(\mathrm{k})}}}= \frac{({\upomega }_{{\mathrm{C}}_{(\mathrm{k}+1)}}^{\mathrm{l}}, {\upomega }_{{\mathrm{C}}_{(\mathrm{k}+1)}}^{\mathrm{m}},{\upomega }_{{\mathrm{C}}_{(\mathrm{k}+1)}}^{\mathrm{u}})}{({\upomega }_{{\mathrm{C}}_{(\mathrm{k})}}^{\mathrm{l}}, {\upomega }_{{\mathrm{C}}_{(\mathrm{k})}}^{\mathrm{m}},{\upomega }_{{\mathrm{C}}_{(\mathrm{k})}}^{\mathrm{u}})}$$

Note that ${\mathrm{\varphi }}_{\mathrm{k}/(\mathrm{k}+1)}$ shows the importance that the criterion of ${\mathrm{C}}_{(\mathrm{k})}$ rank has with respect to the criterion of ${\mathrm{C}}_{(\mathrm{k}+1)}$ rank. Finally, a fuzzy vector of the comparative significance of the evaluation criteria is determined as follows:

10 $$\mathrm{\vartheta }=({\mathrm{\varphi }}_{1/2}, {\mathrm{\varphi }}_{2/3}, \dots {\mathrm{\varphi }}_{\text{k}/{\text{k}+1}})$$

**Step 3.** Next, the fuzzy optimal weights are computed. The final weight values must satisfy two conditions mentioned below:

**Condition 1:** The ratio of weight coefficients of the criteria should be tantamount to their comparative significance:

11 $${\mathrm{\varphi }}_{\frac{\mathrm{k}}{\mathrm{k}+1}}=\frac{{\mathrm{w}}_{\mathrm{k}}}{{\mathrm{w}}_{\mathrm{k}+1}}$$

**Condition 2:** the final weight values should satisfy transitivity regulation as follows:

12 $${\mathrm{\varphi }}_{\mathrm{k}/(\mathrm{k}+1)}\otimes {\mathrm{\varphi }}_{(\mathrm{k}+1)/(\mathrm{k}+2)} =\frac{{\mathrm{w}}_{\mathrm{k}}}{{\mathrm{w}}_{\mathrm{k}+2}}$$

According to the two conditions mentioned above, the final nonlinear model for calculating the optimal fuzzy values of the weight coefficients for all criteria is developed as follows:

min $$\stackrel{\sim }{\upvarepsilon }$$

13 $$s.t\left\{\begin{array}{l}\left|\frac{{\mathrm{w}}_{\mathrm{k}}}{{\mathrm{w}}_{\mathrm{k}+1}}-{\mathrm{\varphi }}_{\mathrm{k}/(\mathrm{k}+1)}\right|\le \stackrel{\sim }{\upvarepsilon } \\ \begin{array}{l} \left|\frac{{\mathrm{w}}_{\mathrm{k}}}{{\mathrm{w}}_{\mathrm{k}+2}}- {\mathrm{\varphi }}_{\mathrm{k}/(\mathrm{k}+1)}\otimes {\mathrm{\varphi }}_{(\mathrm{k}+1)/(\mathrm{k}+2)}\right|\le \stackrel{\sim }{\upvarepsilon }\\ \begin{array}{l}\sum_{\mathrm{j}=1}^{\mathrm{n}}\mathrm{R}\left({\stackrel{\sim }{\mathrm{W}}}_{\mathrm{j}}\right)=1 \\ \begin{array}{l}{\mathrm{l}}_{\mathrm{j}}^{\mathrm{w}}\le {\mathrm{m}}_{\mathrm{j}}^{\mathrm{w}}\le {\mathrm{u}}_{\mathrm{j}}^{\mathrm{w}}\\ {\mathrm{l}}_{\mathrm{j}}^{\mathrm{w}}\ge 0 \end{array}\end{array}\end{array}\\ j=1, 2, \dots , n\end{array}\right.$$

By solving the model mentioned in Eq. (13), the optimal weights $\left({\stackrel{\sim }{\mathrm{w}}}_{1}^{*},{\stackrel{\sim }{\mathrm{w}}}_{2}^{*},\dots ,{\stackrel{\sim }{\mathrm{w}}}_{\mathrm{n}}^{*}\right)$ will be computed. Also, the value of $\upvarepsilon$ shows the deviation from full consistency.

### Fuzzy Measurement Alternatives and Ranking according to the Compromise Solution Method (FMARCOS)

The measurement alternatives and ranking according to the compromise solution (MARCOS) method is initially proposed by Stević et al. as a novel prioritization technique based on the distance of alternatives from the ideal solution and the anti-ideal solution [40]. Compare to other multicriteria decision-making techniques, MARCOS has the advantages of suggesting a new way to calculate utility functions by considering an anti-ideal and an ideal solution simultaneously and providing a closer determination of the utility degree in relation to both solutions [40]. Furthermore, Stanković et al. combined the fuzzy set theory and MARCOS method to deal with the ambiguity and uncertainty of the judgments [32]. According to them, the steps of FMARCOS are as follows:

Step1. Similar to other prioritization techniques, an initial decision-making matrix consisting of n criteria and m alternatives will be developed in the first step. The initial decision-making matrix using the linguistic terms provided in Table 3.

**Table 3:** Linguistic terms for fuzzy MARCOS (Source: [32])

Linguistic terms		Fuzzy numbers
Extremely Poor	EP	(1,1,1)
Very Poor	VP	(1,1,3)
Poor	P	(1,3,3)
Medium Poor	MP	(3,3,5)
Medium	M	(3,5,5)
Medium Good	MG	(5,5,7)
Good	G	(5,7,7)
Very Good	VG	(7,7,9)
Extremely Good	EG	(7,9,9)

14 $$\begin{array}{c}\begin{array}{c}\\ {\mathrm{A}}_{1}\end{array}\\ \begin{array}{c}{\mathrm{A}}_{2}\\ \vdots \end{array}\\ {\mathrm{A}}_{\mathrm{m}}\end{array}\left|\begin{array}{ccc}\begin{array}{c}\begin{array}{c}{\mathrm{C}}_{1}\\ {\stackrel{\sim }{\mathrm{x}}}_{11}\end{array}\\ {\stackrel{\sim }{\mathrm{x}}}_{21}\end{array}& \begin{array}{c}\begin{array}{c}{\mathrm{C}}_{2}\\ {\stackrel{\sim }{\mathrm{x}}}_{12}\end{array}\\ {\stackrel{\sim }{\mathrm{x}}}_{22}\end{array}& \begin{array}{c}\begin{array}{c}\begin{array}{cc}\cdots & {\mathrm{C}}_{\mathrm{n}}\end{array}\\ \begin{array}{cc}\cdots & {\stackrel{\sim }{\mathrm{x}}}_{1\mathrm{n}}\end{array}\end{array}\\ \begin{array}{cc}\cdots & {\stackrel{\sim }{\mathrm{x}}}_{2\mathrm{n}}\end{array}\end{array}\\ \vdots & \vdots & \begin{array}{cc}\ddots & \vdots \end{array}\\ {\stackrel{\sim }{\mathrm{x}}}_{\mathrm{m}1}& {\stackrel{\sim }{\mathrm{x}}}_{\mathrm{m}2}& \begin{array}{cc}\cdots & {\stackrel{\sim }{\mathrm{x}}}_{\mathrm{mn}}\end{array}\end{array}\right|$$

Step 2. Next, an extended initial fuzzy matrix will be constructed by determining the fuzzy anti-ideal $\stackrel{\sim }{\mathrm{A}}(\mathrm{AI})$ and fuzzy ideal $\stackrel{\sim }{\mathrm{A}}(\mathrm{ID})$ solution.

15 $$\begin{array}{c}\begin{array}{c}\begin{array}{c}\\ \stackrel{\sim }{\mathrm{A}}(\mathrm{AI})\end{array}\\ {\stackrel{\sim }{\mathrm{A}}}_{1}\end{array}\\ \begin{array}{c}{\stackrel{\sim }{\mathrm{A}}}_{2}\\ \vdots \end{array}\\ \begin{array}{c}{\stackrel{\sim }{\mathrm{A}}}_{\mathrm{m}}\\ \stackrel{\sim }{\mathrm{A}}(\mathrm{ID})\end{array}\end{array}\left|\begin{array}{ccc}\begin{array}{c}\begin{array}{c}{\mathrm{C}}_{1}\\ \begin{array}{c}{\stackrel{\sim }{\mathrm{x}}}_{\mathrm{ai}1}\\ {\stackrel{\sim }{\mathrm{x}}}_{11}\end{array}\end{array}\\ {\stackrel{\sim }{\mathrm{x}}}_{21}\end{array}& \begin{array}{c}\begin{array}{c}{\mathrm{C}}_{2}\\ \begin{array}{c}{\stackrel{\sim }{\mathrm{x}}}_{\mathrm{ai}2}\\ {\stackrel{\sim }{\mathrm{x}}}_{12}\end{array}\end{array}\\ {\stackrel{\sim }{\mathrm{x}}}_{22}\end{array}& \begin{array}{c}\begin{array}{c}\begin{array}{cc}\cdots & {\mathrm{C}}_{\mathrm{n}}\end{array}\\ \begin{array}{cc}\begin{array}{c}\cdots \\ \cdots \end{array}& \begin{array}{c}{\stackrel{\sim }{\mathrm{x}}}_{\mathrm{ain}}\\ {\stackrel{\sim }{\mathrm{x}}}_{1\mathrm{n}}\end{array}\end{array}\end{array}\\ \begin{array}{cc}\cdots & {\stackrel{\sim }{\mathrm{x}}}_{2\mathrm{n}}\end{array}\end{array}\\ \vdots & \vdots & \begin{array}{cc}\ddots & \vdots \end{array}\\ \begin{array}{c}{\stackrel{\sim }{\mathrm{x}}}_{\mathrm{m}1}\\ {\stackrel{\sim }{\mathrm{x}}}_{\mathrm{id}1}\end{array}& \begin{array}{c}{\stackrel{\sim }{\mathrm{x}}}_{\mathrm{m}2}\\ {\stackrel{\sim }{\mathrm{x}}}_{\mathrm{id}2}\end{array}& \begin{array}{cc}\begin{array}{c}\cdots \\ \cdots \end{array}& \begin{array}{c}{\stackrel{\sim }{\mathrm{x}}}_{\mathrm{mn}}\\ {\stackrel{\sim }{\mathrm{x}}}_{\mathrm{idn}}\end{array}\end{array}\end{array}\right|$$

$\stackrel{\sim }{\mathrm{A}}\left(\mathrm{AI}\right)$ is the anti-ideal or the worst alternative and $\stackrel{\sim }{\mathrm{A}}\left(\mathrm{ID}\right)$ is the ideal or the alternative with the best performance. $\stackrel{\sim }{\mathrm{A}}\left(\mathrm{AI}\right)$ and $\stackrel{\sim }{\mathrm{A}}(\mathrm{ID})$ are defined as follows:

For benefit criteria:

16 $$\stackrel{\sim }{\mathrm{A}}\left(\mathrm{AI}\right)=\underset{\mathrm{i}}{\mathrm{min}}{\stackrel{\sim }{\mathrm{x}}}_{\mathrm{ij}}$$

17 $$\stackrel{\sim }{\mathrm{A}}\left(\mathrm{ID}\right)=\underset{\mathrm{i}}{\mathrm{max}}{\stackrel{\sim }{\mathrm{x}}}_{\mathrm{ij}}$$

For cost criteria:

18 $$\stackrel{\sim }{\mathrm{A}}\left(\mathrm{AI}\right)=\underset{\mathrm{i}}{\mathrm{max}}{\stackrel{\sim }{\mathrm{x}}}_{\mathrm{ij}}$$

19 $$\stackrel{\sim }{\mathrm{A}}\left(\mathrm{ID}\right)=\underset{\mathrm{i}}{\mathrm{min}}{\stackrel{\sim }{\mathrm{x}}}_{\mathrm{ij}}$$

**Step 3.** Afterward, the extended initial fuzzy matrix will be normalized using the following equations:

20 $${\stackrel{\sim }{\mathrm{n}}}_{\mathrm{ij}}=\left({\mathrm{n}}_{{\mathrm{l}}_{\mathrm{ij}}}, {\mathrm{n}}_{{\mathrm{m}}_{\mathrm{ij}}}, {\mathrm{n}}_{{\mathrm{u}}_{\mathrm{ij}}}\right)= \left(\frac{{\mathrm{x}}_{{\mathrm{l}}_{\mathrm{ij}}}}{{\mathrm{x}}_{{\mathrm{u}}_{\mathrm{id}}}} , \frac{{\mathrm{x}}_{{\mathrm{m}}_{\mathrm{ij}}}}{{\mathrm{x}}_{{\mathrm{m}}_{\mathrm{id}}}} ,\frac{{\mathrm{x}}_{{\mathrm{u}}_{\mathrm{ij}}}}{{\mathrm{x}}_{{\mathrm{l}}_{\mathrm{id}}}}\right),\quad \mathrm{ if \,j \,is \,benefit \,criteria}$$

21 $${\stackrel{\sim }{\mathrm{n}}}_{\mathrm{ij}}=\left({\mathrm{n}}_{{\mathrm{l}}_{\mathrm{ij}}}, {\mathrm{n}}_{{\mathrm{m}}_{\mathrm{ij}}}, {\mathrm{n}}_{{\mathrm{u}}_{\mathrm{ij}}}\right)= \left(\frac{{\mathrm{x}}_{{\mathrm{l}}_{\mathrm{id}}}}{{\mathrm{x}}_{{\mathrm{u}}_{\mathrm{ij}}}} , \frac{{\mathrm{x}}_{{\mathrm{m}}_{\mathrm{id}}}}{{\mathrm{x}}_{{\mathrm{m}}_{\mathrm{ij}}}} ,\frac{{\mathrm{x}}_{{\mathrm{u}}_{\mathrm{id}}}}{{\mathrm{x}}_{{\mathrm{l}}_{\mathrm{ij}}}}\right),\quad \mathrm{if \,j \,is \,cost \,criteria}$$

**Step 4.** The normalized extended initial fuzzy matrix will be then multiplied with the fuzzy weight coefficients of the criterion ${\stackrel{\sim }{\mathrm{w}}}_{\mathrm{j}}$ to develop the weighted fuzzy matrix $\stackrel{\sim }{\mathrm{V}}={{[\stackrel{\sim }{\mathrm{ v}}}_{\mathrm{ij}}]}_{\mathrm{m}\times \mathrm{n}}$.

22 $${\stackrel{\sim }{\mathrm{v}}}_{\mathrm{ij}}=\left({\mathrm{v}}_{{\mathrm{l}}_{\mathrm{ij}}}, {\mathrm{v}}_{{\mathrm{m}}_{\mathrm{ij}}}, {\mathrm{v}}_{{\mathrm{u}}_{\mathrm{ij}}}\right)={\stackrel{\sim }{\mathrm{n}}}_{\mathrm{ij}}\otimes {\stackrel{\sim }{\mathrm{w}}}_{\mathrm{j}}=({\mathrm{n}}_{{\mathrm{l}}_{\mathrm{ij}}}\times {\mathrm{w}}_{{\mathrm{l}}_{\mathrm{j}}}, {\mathrm{n}}_{{\mathrm{m}}_{\mathrm{ij}}}\times {\mathrm{w}}_{{\mathrm{m}}_{\mathrm{j}}}, {\mathrm{n}}_{{\mathrm{u}}_{\mathrm{ij}}}\times {\mathrm{w}}_{{\mathrm{u}}_{\mathrm{j}}})$$

**Step 5.** Then, a fuzzy matrix of ${\stackrel{\sim }{\mathrm{s}}}_{\mathrm{i}}$ will be calculated using the following equation:

23 $${\stackrel{\sim }{\mathrm{S}}}_{\mathrm{i}}=\sum_{\mathrm{i}=1}^{\mathrm{n}}{\stackrel{\sim }{\mathrm{v}}}_{\mathrm{ij}}$$

where ${\stackrel{\sim }{\mathrm{s}}}_{\mathrm{i}}({\mathrm{s}}_{\mathrm{i}}^{\mathrm{l}},{\mathrm{s}}_{\mathrm{i}}^{\mathrm{m}},{\mathrm{s}}_{\mathrm{i}}^{\mathrm{u}})$ represent the sum of the elements of the weighted fuzzy matrix $\stackrel{\sim }{\mathrm{V}}$.

**Step 6.** Using the following equations, the utility degree of alternatives ${\stackrel{\sim }{\mathrm{k}}}_{\mathrm{ i}}$ will be determined

24 $${{\stackrel{\sim }{\mathrm{k}}}{_{\mathrm{i}}^{+}}}= \frac{{\stackrel{\sim }{\mathrm{S}}}_{\mathrm{i}}}{{\stackrel{\sim }{\mathrm{S}}}_{\mathrm{id}}}=\left(\frac{{\mathrm{S}}_{{\mathrm{l}}_{\mathrm{i}}}}{{\mathrm{S}}_{{\mathrm{u}}_{\mathrm{id}}}} ,\frac{{\mathrm{S}}_{{\mathrm{m}}_{\mathrm{i}}}}{{\mathrm{S}}_{{\mathrm{m}}_{\mathrm{id}}}} ,\frac{{\mathrm{S}}_{{\mathrm{u}}_{\mathrm{i}}}}{{\mathrm{S}}_{{\mathrm{l}}_{\mathrm{id}}}}\right)$$

25 $${{\stackrel{\sim }{\mathrm{k}}}{_{\mathrm{i}}^{-}}}= \frac{{\stackrel{\sim }{\mathrm{S}}}_{\mathrm{i}}}{{\stackrel{\sim }{\mathrm{S}}}_{\mathrm{ai}}}=\left(\frac{{\mathrm{S}}_{{\mathrm{l}}_{\mathrm{i}}}}{{\mathrm{S}}_{{\mathrm{u}}_{\mathrm{ai}}}} ,\frac{{\mathrm{S}}_{{\mathrm{m}}_{\mathrm{i}}}}{{\mathrm{S}}_{{\mathrm{m}}_{\mathrm{ai}}}} ,\frac{{\mathrm{S}}_{{\mathrm{u}}_{\mathrm{i}}}}{{\mathrm{S}}_{{\mathrm{u}}_{\mathrm{ai}}}}\right)$$

**Step 7.** In the next step, the fuzzy matrix ${\stackrel{\sim }{\mathrm{T}}}_{\mathrm{i}}$ is developed using the following equation:

26 $${\stackrel{\sim }{\mathrm{T}}}_{\mathrm{i}}={\stackrel{\sim }{\mathrm{t}}}_{\mathrm{i}}=\left({\mathrm{t}}_{{\mathrm{l}}_{\mathrm{i}}}, {\mathrm{t}}_{{\mathrm{m}}_{\mathrm{i}}}, {\mathrm{t}}_{{\mathrm{u}}_{\mathrm{i}}}\right)={{\stackrel{\sim }{\mathrm{k}}}{_{\mathrm{i}}^{+}}}+{{\stackrel{\sim }{\mathrm{k}}}{_{\mathrm{i}}^{+}}}=({{\mathrm{k}}{_{{\mathrm{l}}_{\mathrm{i}}}^{-+}}}+{{\mathrm{k}}{_{{\mathrm{l}}_{\mathrm{i}}}^{-}}}, {{\mathrm{k}}{_{{\mathrm{m}}_{\mathrm{i}}}^{+}}}+{{\mathrm{k}}{_{{\mathrm{m}}_{\mathrm{i}}}^{-}}}, {{\mathrm{k}}^{+}}_{{\mathrm{u}}_{\mathrm{i}}}+{{\mathrm{k}}{_{{\mathrm{u}}_{\mathrm{i}}}^{-}}})$$

Then, it is necessary to determine a new fuzzy number $\stackrel{\sim }{\mathrm{D}}$, and defuzzify it according to Eq. (2) to determine the value of ${\mathrm{df}}_{\mathrm{crisp}}$.

27 $$\stackrel{\sim }{\mathrm{D}}=\left({\mathrm{d}}_{\mathrm{l}},{\mathrm{d}}_{\mathrm{m}},{\mathrm{d}}_{\mathrm{u}}\right)=\underset{\mathrm{i}}{\mathrm{max}}{\stackrel{\sim }{\mathrm{t}}}_{\mathrm{ij}}$$

**Step 8.** The utility functions in relation to the ideal $\mathrm{f}({\stackrel{\sim }{\mathrm{K}}}{_{\mathrm{i}}^{+}})$ and anti-ideal $\mathrm{f}({\stackrel{\sim }{\mathrm{K}}}{_{\mathrm{i}}^{+}})$ solution is calculated as follows:

28 $$\mathrm{f}\left({{\stackrel{\sim }{\mathrm{k}}}{_{\mathrm{i}}^{+}}}\right)=\frac{{{\stackrel{\sim }{\mathrm{k}}}{_{\mathrm{i}}^{-}}}}{{\mathrm{df}}_{\mathrm{crisp}}}=\left(\frac{{{\mathrm{k}}^{-}_{{\mathrm{l}}_{\mathrm{i}}}}}{{\mathrm{df}}_{\mathrm{crisp}}}, \frac{{{\mathrm{k}}^{-}_{{\mathrm{m}}_{\mathrm{i}}}}}{{\mathrm{df}}_{\mathrm{crisp}}} ,\frac{{{\mathrm{k}}^{-}_{{\mathrm{u}}_{\mathrm{i}}}}}{{\mathrm{df}}_{\mathrm{crisp}}}\right)$$

29 $$\mathrm{f}\left({{\stackrel{\sim }{\mathrm{k}}}{_{\mathrm{i}}^{-}}}\right)=\frac{{{\stackrel{\sim }{\mathrm{k}}}{_{\mathrm{i}}^{+}}}}{{\mathrm{df}}_{\mathrm{crisp}}}=\left(\frac{{{\mathrm{k}}^{+}_{{\mathrm{l}}_{\mathrm{i}}}}}{{\mathrm{df}}_{\mathrm{crisp}}}, \frac{{{\mathrm{k}}^{+}_{{\mathrm{m}}_{\mathrm{i}}}}}{{\mathrm{df}}_{\mathrm{crisp}}} ,\frac{{{\mathrm{k}}^{+}_{{\mathrm{u}}_{\mathrm{i}}}}}{{\mathrm{df}}_{\mathrm{crisp}}}\right)$$

**Step 9.** Ultimately, the utility function of alternatives $\mathrm{f}\left({\mathrm{k}}_{\mathrm{i}}\right)$ is calculated as follows:

30 $$\mathrm{f}\left({\mathrm{k}}_{\mathrm{i}}\right)=\frac{{{\mathrm{k}}{_{\mathrm{i}}^{+}}}+{{\mathrm{k}}{_{\mathrm{i}}^{-}}}}{1+\frac{1-\mathrm{f}({{\mathrm{k}}{_{\mathrm{i}}^{+}}})}{\mathrm{f}({{\mathrm{k}}{_{\mathrm{i}}^{+}}})}+\frac{1-\mathrm{f}({{\mathrm{k}}{_{\mathrm{i}}^{-}}})}{\mathrm{f}({{\mathrm{k}}{_{\mathrm{i}}^{-}}})}}$$

The alternatives are prioritized according to the value of utility function $\mathrm{f}\left({\mathrm{k}}_{\mathrm{i}}\right)$, The highest value of the utility function shows the best alternative.

## Results

### Developing an MCDM model

In order to identify the criteria for selecting the most appropriate organizational modalities, a systematic literature review was conducted. To this end, five major scientific databases namely, Web of Science, Scopus, PubMed, Embase, and Cochrane database as well as grey resources were searched to find the relevant articles using different keywords including structural adjustment, hospital, and healthcare. However, the search strings specific to each database are as follows:

Web of Science:

TOPIC: ((autonom* OR corporat* OR privat* OR "structural adjustment") AND ("efficiency" OR "satisfaction") AND ("hospital" OR "healthcare")).

Scopus:

TITLE-ABS-KEY ((autonom* OR corporat* OR privat* OR "structural adjustment") AND ("efficiency" OR "satisfaction") AND ("hospital" OR "healthcare")).

PubMed:

(autonom* OR corporat* OR privat* OR "structural adjustment") AND ("efficiency" OR "satisfaction") AND ("hospital" OR "healthcare").

Embase:

autonom* OR corporat* OR privat* OR 'structural adjustment' AND ('efficiency'/exp OR 'efficiency' OR 'job satisfaction'/exp OR 'job satisfaction') AND ('hospital'/exp OR 'hospital' OR 'healthcare'/exp OR 'healthcare').

Cochrane:

(autonom* OR corporat* OR privat* OR "structural adjustment") AND ("efficiency" OR "satisfaction") AND ("hospital" OR "healthcare").

**Fig. 2:**
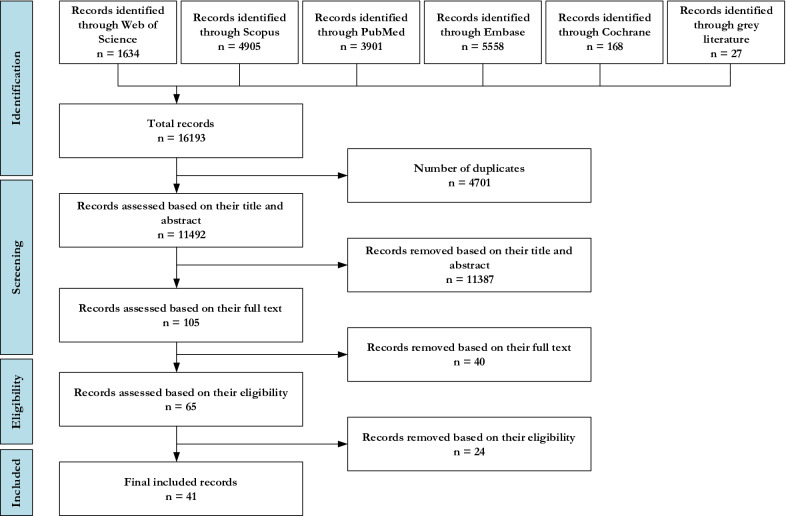
Systematic literature review process

The search was limited to records published in the English language in the period between 1985 to 2022. Also, their quality was evaluated according to the NICE checklist [41]. The result of the systematic review was a total number of 16,193 records, which were screened based on their title, abstract, and the quality of their context. Finally, 41 articles were selected for identifying the pertinent criteria. Figure 2 shows the screening process of the systematic review. Also, the criteria identified through systematic review are available in Table 4.

**Table 4:** Criteria for selecting the best organizational modalities in hospitals

Criteria		Definition	References
C1	Access	Availability of services for recipients of health services and their ability of patients to receive these services	[42–45]
C2	Hospital admissions	The number of patients admitted by the hospital during a specific period	[46–48]
C3	Average Length of Stay(ALOS)	The number of days the patient spends in the hospital since admission is divided by the number of people discharged (including deaths during the year)	[48–50]
C4	Outpatient visits	The number of outpatients referred to the hospital during a specific period	[51, 52]
C5	Bed occupancy rate	The number of beds used by the hospital in a certain period compared to all the beds in the hospital	[52, 53]
C6	Income	The amount of financial income earned by the hospital during a certain period	[45, 48, 54–59]
C7	Number of personnel	Number of staff in the hospital	[61–63]
C8	Status of equipment	The level of relative quality and modernity of the hospital equipment	[4, 64]
C9	Patient satisfaction	The level of patients' satisfaction with the services received from the hospital	[65, 66]
C10	Employee satisfaction	The level of satisfaction of hospital staff from the organizational unit in which they are employed	[67–69]

**Fig. 3:**
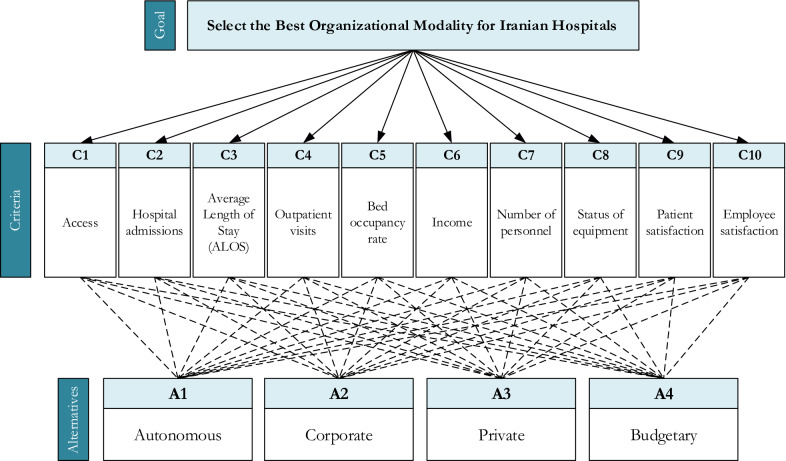
Research MCDM model

The results of the systematic literature review and the four organizational modalities mentioned earlier helped to develop an MCDM model, which is illustrated in Fig. 3.

### Calculating the weights of criteria

To prioritize the organizational modalities, it is necessary to first calculate the weights of the identified criteria. As previously stated, in the present study FUCOM-F was employed to determine the weights. To this end, 6 DMs (mentioned in Table 1) were asked to provide the initial rank of the criteria based on their judgment. Next, the DMs mutually compared the most significant criteria based on the fuzzy linguistic term provided in Table 2. Then, the fuzzy criterion significance was determined according to the mutual pairwise comparisons and Eq. (9). In the next step, 6 nonlinear models were developed for each DM using the two conditions mentioned in Eqs. (11) and (12). The models were solved using LINGO 18.0 software to obtain the optimal weights. Table 5 shows the weights and deviation from full consistency (ε) for each DM.

**Table 5:** Fuzzy weights of criteria for each decision maker

Criteria	DMs		
	E1	E2	E3
C1	(0.0569, 0.0720, 0.0953)	(0.1586, 0.1590, 0.1590)	(0.0437, 0.0513, 0.0610)
C2	(0.0954, 0.1436, 0.2131)	(0.0354, 0.0404, 0.0458)	(0.0438, 0.0514, 0.0613)
C3	(0.0958, 0.1436, 0.2135)	(0.0455, 0.0534, 0.0635)	(0.0610, 0.0769, 0.1017)
C4	(0.0410, 0.0482, 0.0572)	(0.0354, 0.0403, 0.0456)	(0.0608, 0.0767, 0.1017)
C5	(0.0950, 0.1439, 0.2121)	(0.0633, 0.0799, 0.1059)	(0.0340, 0.0388, 0.0440)
C6	(0.1430, 0.1434, 0.1434)	(0.0633, 0.0798, 0.1059)	(0.1523, 0.1527, 0.1527)
C7	(0.0952, 0.1439, 0.2126)	(0.1058, 0.1593, 0.2363)	(0.1020, 0.1530, 0.2274)
C8	(0.0318, 0.0361, 0.0408)	(0.1056, 0.1595, 0.2358)	(0.1014, 0.1532, 0.2264)
C9	(0.0569, 0.0720, 0.0953)	(0.1062, 0.1593, 0.2368)	(0.1016, 0.1530, 0.2269)
C10	(0.0318, 0.0364, 0.0411)	(0.0454, 0.0534, 0.0635)	(0.0608, 0.0766, 0.1017)
ε	0.00023	0.00026	0.00025

Criteria	DMs		
	E4	E5	E6
C1	(0.1543, 0.1547, 0.1547)	(0.1586, 0.1590, 0.1590)	(0.0569, 0.0720, 0.0953)
C2	(0.1030, 0.1550, 0.2299)	(0.0633, 0.0798, 0.1059)	(0.0954, 0.1436, 0.2131)
C3	(0.0618, 0.0779, 0.1030)	(0.0455, 0.0534, 0.0635)	(0.0958, 0.1436, 0.2135)
C4	(0.0443, 0.0520, 0.0618)	(0.0354, 0.0403, 0.0456)	(0.0410, 0.0482, 0.0572)
C5	(0.0616, 0.0777, 0.1030)	(0.0633, 0.0799, 0.1059)	(0.0950, 0.1439, 0.2121)
C6	(0.1033, 0.1550, 0.2304)	(0.1058, 0.1593, 0.2363)	(0.1430, 0.1434, 0.1434)
C7	(0.0344, 0.0391, 0.0443)	(0.0354, 0.0404, 0.0458)	(0.0952, 0.1439, 0.2126)
C8	(0.0345, 0.0393, 0.0445)	(0.1056, 0.1595, 0.2358)	(0.0318, 0.0361, 0.0408)
C9	(0.1028, 0.1552, 0.2294)	(0.1062, 0.1593, 0.2368)	(0.0569, 0.0720, 0.0953)
C10	(0.0616, 0.0776, 0.1030)	(0.0454, 0.0534, 0.0635)	(0.0318, 0.0364, 0.0411)
ε	0.00025	0.00026	0.00023

**Fig. 4:**
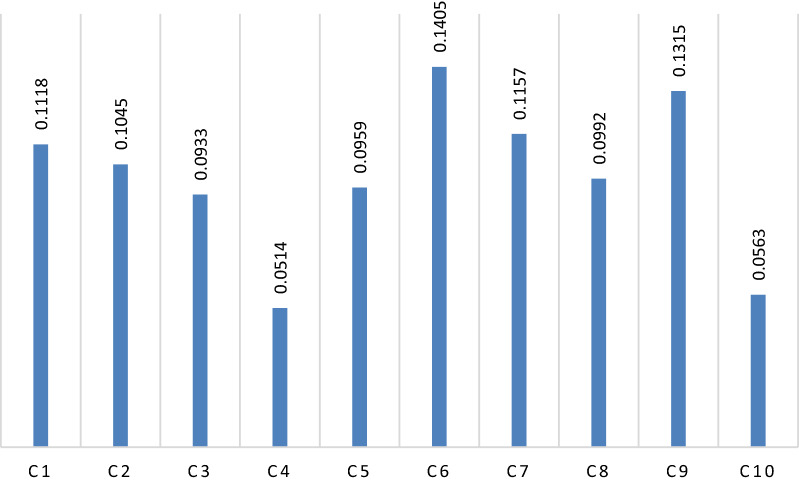
Crisp weights of criteria

The individual judgments are then aggregated to determine a single weight vector. The most common technique to aggregate individual judgments is the arithmetic mean [70]. Therefore, an arithmetic mean was used to obtain the final fuzzy weights. Even though the final fuzzy weights were used for ranking the alternatives in the next phase, final fuzzy weights were also transformed into crisp ones for better discussion on the criteria. Table 6 demonstrates the final fuzzy weights, final crisp weight, and ranking of the criteria, Fig. 4 depicts the final crisp weight as well.

**Table 6:** Final weights and ranking of the criteria

Criteria	Fuzzy weights	Crisp weights	Ranking
Access (C1)	(0.1048, 0.1114, 0.1207)	0.1118	4
Hospital admissions (C2)	(0.0727, 0.1023, 0.1448)	0.1045	5
Average Length of Stay(ALOS) (C3)	(0.0676, 0.0915, 0.1265)	0.0933	8
Outpatient visits (C4)	(0.0430, 0.0510, 0.0615)	0.0514	10
Bed occupancy rate (C5)	(0.0687, 0.0940, 0.1305)	0.0959	7
Income (C6)	(0.1185, 0.1389, 0.1687)	0.1405	1
Number of personnel (C7)	(0.0780, 0.1132, 0.1632)	0.1157	3
Status of equipment (C8)	(0.0685, 0.0973, 0.1374)	0.0992	6
Patient satisfaction (C9)	(0.0884, 0.1285, 0.1867)	0.1315	2
Employee satisfaction (C10)	(0.0461, 0.0556, 0.0690)	0.0563	9

According to Fig. 4, income (C6) is the most important criterion for selecting the most appropriate organizational modality in Iranian hospitals, with a weight coefficient of 0.1405. Moreover, patient satisfaction (C9), number of personnel (C7), access (C1), hospital admissions (C2), status of equipment (C8), bed occupancy rate (C5), the average length of stay (ALOS) (C3), and employee satisfaction (C10) are the most vital factors, respectively. Also, outpatient visits (C4) have the lowest significance with a weight coefficient of only 0.0514. This result of this study is consistent with various previous research that investigated the effect of organizational modalities and structural adjustment policies in the health sector, particularly in hospitals. Findings of Studies conducted by Collins et al. [51], Sharma and Hotchkiss [56], Shen [47], Kim and McCue [57], Huang et al. [67], Maharani et al. [53], Pan et al. [58], and Maharani and Tampubolon [64] indicate that revenue or income of the healthcare providers such as hospitals is the most influential factor for selecting structural adjustment policies. However, some studies such as Maruthappu et al. and Jones and Kantarjian mention that health must be considered as a public right and all the population has to access to health services equally; therefore, no attention must be paid to the financial aspects of healthcare services, especially in hospitals [71, 72].

### Prioritizing the organizational modalities

According to the MCDM model presented in Fig. 3, there are four organizational modalities for hospitals, which are autonomous, corporate, private, and budgetary. To prioritize these policies using FMARCOS, a questionnaire was handed out to DMs. They were asked to express the preferences of each policy with respect to criteria based on the fuzzy linguistic terms in Table 3 to form the initial fuzzy matrix. Also, it should be noted that arithmetic mean was adopted to aggregate the initial fuzzy matrix for each expert into a single one since it is the most common aggregation method [70]. Then, the extended initial fuzzy matrix was developed using Eqs. (16) to (19). Noteworthy to mention that access (C1), hospital admissions (C2), outpatient visits (C3), income (C4), the status of equipment (C8), patient satisfaction (C9), and employee satisfaction (C10) are benefit criteria. In contrast, the average length of stay (C3), bed occupancy rate (C5), and the number of personnel (C7) belong to the cost criteria group. After normalizing the extended initial fuzzy matrix using Eqs. (20) and (21), the weighted fuzzy matrix was developed by multiplying the normalized extended initial fuzzy matrix with the fuzzy weights determined by FUCOM-F. In the following, the aggregated initial fuzzy matrix, the extended initial fuzzy matrix, normalized extended initial fuzzy matrix, and weighted fuzzy matrix are shown in Tables 7, 8, 9, 10, respectively.

**Table 7:** Aggregated initial fuzzy matrix

		Criteria			
		C1	C2	…	C10
Alternatives	A1	(2.6667, 4.3333, 4.6667)	(2.6667, 3.6667, 4.6667)	…	(4.0000, 4.6667, 5.6667)
	A2	(2.3333, 3.6667, 4.3333)	(3.0000, 3.6667, 5.0000)	…	(3.0000, 3.6667, 4.6667)
	A3	(4.3333, 5.6667, 6.3333)	(4.6667, 5.6667, 6.6667)	…	(5.0000, 6.3333, 7.0000)
	A4	(1.3333, 2.0000,2.0000)	(2.6667, 3.6667, 4.0000)	…	(4.6667, 6.0000, 6.6667)

**Table 8:** Extended initial fuzzy matrix

		Criteria			
		C1	C2	…	C10
Alternatives	AI	(1.3333, 2.0000, 2.0000)	(2.6667, 3.6667, 4.0000)	…	(3.0000, 3.6667, 4.6667)
	A1	(2.6667, 4.3333, 4.6667)	(2.6667, 3.6667, 4.6667)	…	(4.0000, 4.6667, 5.6667)
	A2	(2.3333, 3.6667, 4.3333)	(3.0000, 3.6667, 5.0000)	…	(3.0000, 3.6667, 4.6667)
	A3	(4.3333, 5.6667, 6.3333)	(4.6667, 5.6667, 6.6667)	…	(5.0000, 6.3333, 7.0000)
	A4	(1.3333, 2.0000,2.0000)	(2.6667, 3.6667, 4.0000)	…	(4.6667, 6.0000, 6.6667)
	ID	(4.3333, 5.6667, 6.3333)	(4.6667, 5.6667, 6.6667)	…	(5.0000, 6.3333, 7.0000)

**Table 9:** Normalized extended initial fuzzy matrix

		Criteria			
		C1	C2	…	C10
Alternatives	AI	(0.2105, 0.3158, 0.3158)	(0.4000, 0.5500, 0.6000)	…	(0.4286, 0.5238, 0.6667)
	A1	(0.4211, 0.6842, 0.7368)	(0.4000,0.5500, 0.7000)	…	(0.5714, 0.6667, 0.8095)
	A2	(0.3684, 0.5789, 0.6842)	(0.4500, 0.5500, 0.7500)	…	(0.4286, 0.5238, 0.6667)
	A3	(0.6842, 0.8947, 1.0000)	(0.7000, 0.8500, 1.0000)	…	(0.7143, 0.9048, 1.0000)
	A4	(0.2105, 0.3158,0.3158)	(0.4000, 0.5500, 0.6000)	…	(0.6667, 0.8571, 0.9524)
	ID	(0.6842, 0.8947, 1.0000)	(0.7000, 0.8500, 1.0000)	…	(0.7143,0.9048, 1.0000)

**Table 10:** Weighted Fuzzy Matrix

		Criteria			
		C1	C2	…	C10
Alternatives	AI	(0.0221, 0.0352, 0.0381)	(0.0291, 0.0563, 0.0869)	…	(0.0198, 0.0291, 0.0460)
	A1	(0.0441, 0.0762, 0.0889)	(0.0291, 0.0563,0.1014)	…	(0.0264, 0.0371, 0.0559)
	A2	(0.0386, 0.0645, 0.0826)	(0.0327, 0.0563, 0.1086)	…	(0.0198, 0.0291, 0.0460)
	A3	(0.0717, 0.0996, 0.1207)	(0.0509, 0.0870, 0.1448)	…	(0.0329, 0.0503, 0.0690)
	A4	(0.0221, 0.0352, 0.0381)	(0.0291, 0.0563, 0.0869)	…	(0.0308, 0.0477, 0.0657)
	ID	(0.0717, 0.0996, 0.1207)	(0.0509, 0.0870, 0.1448)	…	(0.0329, 0.0503, 0.0690)

In the next step, the matrix ${\stackrel{\sim }{\mathrm{s}}}_{\mathrm{i}}$,${{\stackrel{\sim }{\mathrm{k}}}{_{\mathrm{i}}^{+}}}$, ${{\stackrel{\sim }{\mathrm{k}}}{_{\mathrm{i}}^{-}}}$ and ${\stackrel{\sim }{\mathrm{T}}}_{\mathrm{i}}$ are calculated using Eqs. (23) to (26), respectively. Table 11 shows these matrices for each alternative

**Table 11:** Matrix ${\stackrel{\sim }{\mathbf{s}}}_{\mathbf{i}}$,${{\stackrel{\sim }{\mathbf{k}}}{_{\mathbf{i}}^{+}}}$, ${{\stackrel{\sim }{\mathbf{k}}}{_{\mathbf{i}}^{-}}}$ and ${\stackrel{\sim }{\mathbf{T}}}_{\mathbf{i}}$

	${\stackrel{\sim }{\mathrm{s}}}_{\mathrm{i}}$	${{\stackrel{\sim }{\mathrm{k}}}{_{\mathrm{i}}^{+}}}$	${{\stackrel{\sim }{\mathrm{k}}}{_{\mathrm{i}}^{-}}}$	${\stackrel{\sim }{\mathrm{T}}}_{\mathrm{i}}$
AI	(0.3196, 0.5153, 0.7250)			
A1	(0.4603,0.6535, 0.9456)	(0.4015, 0.7894, 1.5786)	(0.6349, 1.2682, 2.9590)	(1.0364, 2.0576, 4.5376)
A2	(0.4565,0.6256, 0.9222)	(0.3981, 0.7557, 1.5395)	(0.6296, 1.2141,2.8858)	(1.0277, 1.9697, 4.4252)
A3	(0.5043, 0.7690, 1.0617)	(0.4399, 0.9289, 1.7724)	(0.6956, 1.4923,3.3225)	(1.1355, 2.4212, 5.0949)
A4	(0.4218, 0.6428, 0.8507)	(0.3679, 0.7765,1.4202)	(0.5818, 1.2475, 2.6622)	(0.9497, 2.0239, 4.0824)
ID	(0.5990, 0.8279, 1.1465)			

According to Eq. (27) a new fuzzy number $\stackrel{\sim }{\mathrm{D}}$ is developed and defuzzified using Eq. (2). The number $\stackrel{\sim }{\mathrm{D}}$ is as follows:

$$\stackrel{\sim }{\mathrm{D}}$$ = (1.1355, 2.4212, 5.0949), and $${\mathrm{df}}_{\mathrm{crisp}}$$= 2.6525

**Fig. 5:**
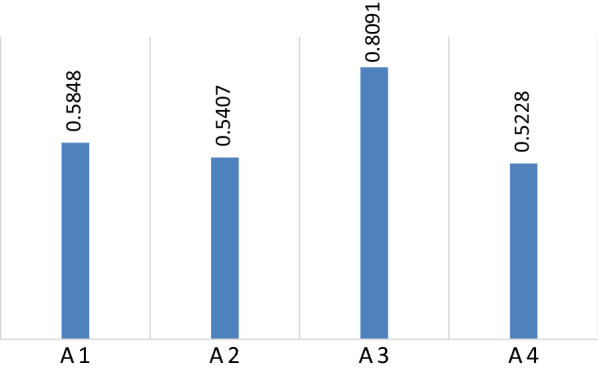
Utility function of alternatives

Finally, the utility functions in relation to the ideal $\mathrm{f}({\stackrel{\sim }{\mathrm{K}}}{_{\mathrm{i}}^{+}})$ and anti-ideal $\mathrm{f}({\stackrel{\sim }{\mathrm{K}}}{_{\mathrm{i}}^{-}})$ solution, and the utility function of alternatives $\mathrm{f}\left({\mathrm{k}}_{\mathrm{i}}\right)$ is calculated using Eqs. (28) to (30), respectively. Needless to say, the highest value of the utility function shows the best alternative. Table 12 shows the utility functions and the final ranking of alternatives. Also, Fig. 5 illustrates the utility functions of each alternative.

**Table 12:** Utility function and ranking of alternatives

Alternative	$\mathrm{f}({\stackrel{\sim }{\mathrm{K}}}{_{\mathrm{i}}^{+}})$	$\mathrm{f}({\stackrel{\sim }{\mathrm{K}}}{_{\mathrm{i}}^{-}})$	$\mathrm{f}\left({\mathrm{k}}_{\mathrm{i}}\right)$	Ranking
Autonomous (A1)	(0.2394, 0.4781, 1.1156)	(0.1514, 0.2976, 0.5951)	0.5848	2
Corporate (A2)	(0.2374, 0.4577, 1.0879)	(0.1501, 0.2849, 0.5804)	0.5407	3
Private A3)	(0.2623, 0.5626, 1.2526)	(0.1658, 0.3502, 0.6682)	0.8091	1
Budgetary (A4)	(0.2193, 0.4703, 1.0036)	(0.1387, 0.2927, 0.5354)	0.5228	4

### Discussion

As depicted by Fig. 5, the private modality (A3) is the most appropriate organizational modality for Iranian hospitals by stark contrast, with the utility function value of 0.8091. A controversial finding which has been under a fierce debate between academics, politicians and ordinary people due to its political nature and more importantly conflicting reports on its effects on efficiency, service quality, equity and access in healthcare sector [73–79]. Despite the existence of conflicting reports on the effects of private structure in hospitals on different aspects of healthcare delivery and financing, our experts shared a mutual view on the issue of private initiative in healthcare sector with international organizations experts and policy makers like world bank and IMF which implicitly shows the influence of such international organizations programs and policies on the healthcare academics in Iran [45, 80–84].

Furthermore, there is a negligible difference amongst organizational modalities, autonomous (A1) and corporate (A2) are considered the best based on the judgment of experts, with the utility function value of 0.5848 and 0.5407, respectively. Moreover, budgetary (A4) is the least appropriate organizational modality, with the utility function value of 0.5228. Such ranking can be decisive for healthcare managers and policy makers during the transitional period of healthcare industry into a market-based entity through restructuring the organizational aspects of the service providers, since according to some studies, restructuring healthcare organizations and the transition of service delivery to market-type mechanisms needs a long-term planning with a precious analysis of organizational environment and warn healthcare managers and politicians of severe costs and backlash if such transition happens without a situational analysis and in a go; A fact that shows such restructuring programs cannot be applied to all organizations with different circumstances with a single framework and more importantly needs to be done in a form of step by step process of restructuring towards a fully private structure providing healthcare services [42, 85, 86].

As mentioned earlier, similar to our findings, implications of numerous previous studies conducted by international organizations such as the World Bank and the International Monetary Fund and other researchers show the suitability of the private modality for hospitals [47, 49, 50, 55, 65, 87] and inadequacy of budgetary and fully governmental organizational modality [58, 88, 89]. However, it`s worthy of mentioning that some studies have also reached different and contradictory conclusions. For instance, Dahlgren, Doshmangir et al., and Pan et al. mentioned that not only hospital performance indicators will not improve by changing the organizational modality of hospitals into a private one, but even in some cases, it causes diminished hospital performance. Nonetheless, the findings of this study are in accordance with the more highlighted point of view, meaning appropriateness of the private modality for hospitals [58, 88, 89]; A phenomenon which clearly demonstrates the conflicting and undecisive results derived from studies on the context; something which led us to use the views of local experts rather than international academics earlier in the study.

### Managerial implications

The results obtained based on the judgments of the experts of this study provide several practical suggestions for the hospital managers and policymakers in Iran and other similar developing countries, who want to apply structural adjustment policies in order to change the organizational modality of their hospitals. As the results of the literature review show, the most crucial recommendation is that if there is a need and also a strong will to make a change, managers and policymakers should better consider a full transition to a private organizational modality rather than shift only to a corporate or an autonomous one. It needs to be mentioned that as several studies have shown, in most cases, specially in developing countries, there is a need to bring upon the structural adjustment initiative in a step by step long-term run due to several factors like socio-economic and geopolitical aspects of each case; otherwise the initiative may result in the opposite of what is aimed for [84, 90]. Furthermore, since there is a possibility that privatization causes an increase in health services costs, hospital managers and policymakers should pay attention to the problems and issues of the public population with lower income ensuring justice in the distribution of health services as a public right.

## Conclusion

The objective of this study was to prioritize the four organizational modalities, namely autonomous, corporate, private, and budgetary for Iranian hospitals. To do so, in the first step, a systematic literature review was conducted to find the respective prioritization criteria and develop a multicriteria decision-making model. Then, the policies were prioritized using MCDM techniques in a fuzzy environment. Integrating Fuzzy sets to MCDM techniques can be very helpful in uncertain decision-making environments by reducing the vagueness, ambiguity, and subjectiveness of the decision-makers (DMs). The adopted techniques were FUCOM-F for determining the coefficient weights and FMARCOS for prioritization of alternatives, which both of them have several advantages compared to other similar techniques. FUCOM has fewer pairwise comparisons and MARCOS considers both anti-ideal and an ideal solution simultaneously providing more reliable results. According to the findings, income is the most vital criterion in selecting organizational modalities for hospitals and the private modality is the most appropriate organizational modality for Iranian hospitals; a fact that clearly demonstrates the growing need of the healthcare sector in Iran to exponentially integrate and comply with market and market type mechanisms either through a direct privatization initiative or a step by step scenario through autonomization or corporatization with an eye to convert hospitals to totally private entities in the long run which is supported by other results of this study which prioritize autonomous and corporate hospitals over budgetary ones [84, 90]. An agenda which is closely aligned with IMF and world bank policies in developing countries like Iran which clearly shows the high capacity in the Iranian healthcare sector to potentially get involved in such international organizations programs and initiatives in the near future. Moreover, this study contributes to the literature by proposing a novel methodology based on FUCOM and MARCOS in a fuzzy environment for the first time in the healthcare management context and helps hospital managers and healthcare policymakers in developing countries regarding organizational modalities and structural adjustment policies. Finally, it should be mentioned that there are some limitations regarding the results of the study due to the possible existence of conflict of interest in the experts which possibly can be in contrast with views of a high amount users in the healthcare system at times due to their arguably higher socioeconomic status than most of the patients using services of hospitals in Iran; a hypothesis which is needed to get addressed in the future research regarding this scope of study. This paper highlights the need for acquiring new approaches in studying and benchmarking organizational modalities in the research area of structural adjustment policies by using a Fuzzy FUCOM-MARCOS Approach as a brand-new initiative to be applied and modeled by upcoming studies related to the literature.

## Data Availability

The datasets used and/or analyzed during the current study are included in the tables of the published article. More data are available from the corresponding author on reasonable request.

## References

[1] Stiglitz J (2002). Globalization and its discontents.

[2] Lensink R (1996). Structural adjustment in sub-Saharan Africa.

[3] Harding A, Preker A (2000). Understanding organizational reforms.

[4] Sepehri A (2014). Does autonomization of public hospitals and exposure to market pressure complement or debilitate social health insurance systems? Evidence from a low-income country. Int J Health Serv.

[5] Audretsch DB, Lehmann E (2016). The seven secrets of Germany.

[6] White H. Adjustment in Africa*.* Development and Change. 1996: 785–815.

[7] Fiedler FE (1987). New approaches to leadership, cognitive resources and organizational performance.

[8] Govindaraj R, Chawla M. Recent experiences with hospital autonomy in developing countries: what can we learn? 1996: Harvard school of public health. Department of population and international health. DDM.

[9] McPake B (2003). Is the Colombian health system reform improving the performance of public hospitals in Bogota?. Health Policy Plan.

[10] Kalhori N., SH, Analyzing the effectiveness and efficiency of the new system of administration of hospitals within hospitals on general hospitals—Iran University of Medical Sciences*.* [Thesis in Persian]. Tehran: Iran University of Medical Sciences, School of Management and Medical Information Sciences, 2005.

[11] Gathron EL. The Corporatization of America's Healthcare System: implications for compassion fatigue among nurses. 2013.

[12] Collyer F, White K (2001). Corporate control of healthcare in Australia.

[13] Kahancová M, Szabó I, Acting on the edge of public sector: Hospital corporatization and collective bargaining in Hungary and Slovakia. GUSTO Project WP, 2012. **6**.

[14] Villa S, Kane N (2013). Assessing the impact of privatizing public hospitals in three American states: implications for universal health coverage. Value Health.

[15] Albreht T (2009). Privatization processes in health care in Europe—a move in the right direction, a ‘trendy’option, or a step back?. Eur J Public Health.

[16] Triantaphyllou E (2000). Multi-criteria decision making methods: a comparative study.

[17] Božanić D (2021). D numberS – FUCOM – fuzzy rafsi model for selecting the group of construction machines for enabling mobility. Facta Universitatis Ser Mech Eng..

[18] Mitrović Simić J (2020). A Novel CRITIC-Fuzzy FUCOM-DEA-Fuzzy MARCOS model for safety evaluation of road sections based on geometric parameters of road. Symmetry.

[19] Bakır M, Atalık Ö (2021). Application of fuzzy AHP and fuzzy MARCOS approach for the evaluation of e-service quality in the airline industry. Decis Making Appl Manag Eng.

[20] Pamucar D, Ecer F, Deveci M, Assessment of alternative fuel vehicles for sustainable road transportation of United States using integrated fuzzy FUCOM and neutrosophic fuzzy MARCOS methodology. (1879–1026 (Electronic)). 10.1016/j.scitotenv.2021.147763 34029824

[21] Stević Ž, Brković N (2020). A novel integrated FUCOM-MARCOS model for evaluation of human resources in a transport company. Logistics.

[22] Blagojević A (2021). Evaluation of safety degree at railway crossings in order to achieve sustainable traffic management: a novel integrated fuzzy MCDM model. Sustainability.

[23] Bozanic D, Tešić D, Milić A (2020). Multicriteria decision making model with Z-numbers based on FUCOM and MABAC model. Decis Making Appl Manag Eng.

[24] Puška A (2020). Evaluation software of project management used measurement of alternatives and ranking according to compromise solution (MARCOS) method. Oper Res Eng Sci Theo Appl.

[25] Biswas S (2020). Measuring performance of healthcare supply chains in India: a comparative analysis of multi-criteria decision making methods. Decis Making.

[26] Durmić E (2020). Sustainable supplier selection using combined FUCOM—rough SAW model. Rep Mech Eng.

[27] Goguen JA, Zadeh LA. Fuzzy sets. Information and control, vol. 8 (1965), pp. 338–353. – Zadeh LA. Similarity relations and fuzzy orderings. Information sciences, vol. 3 (1971), pp. 177–200. Journal of Symbolic Logic, 2014. 38(4): p. 656–657.

[28] Ahmadi H, Nilashi M, Ibrahim O (2015). Organizational decision to adopt hospital information system: an empirical investigation in the case of Malaysian public hospitals. Int J Med Inform.

[29] Si SL (2017). Identifying key performance indicators for holistic hospital management with a modified DEMATEL approach. Int J Environ Res Public Health.

[30] Torkzad A, Beheshtinia MA (2019). Evaluating and prioritizing hospital service quality. Int J Health Care Qual Assur.

[31] Kadoić N (2021). Measuring quality of public hospitals in croatia using a multi-criteria approach. Int J Environ Res Public Health.

[32] Stanković M (2020). A new fuzzy MARCOS method for road traffic risk analysis. Mathematics.

[33] Pamučar D, Stević Ž, Sremac S (2018). A new model for determining weight coefficients of criteria in MCDM models: full consistency method (FUCOM). Symmetry.

[34] Rezaei J (2015). Best-worst multi-criteria decision-making method. Omega.

[35] Saaty TL. Group decision making and the AHP. In The analytic hierarchy process Springer, Berlin, Heidelberg, 1989.

[36] Pamucar D, Ecer F (2020). Prioritizing the weights of the evaluation criteria under fuzziness: the fuzzy full consistency method – FUCOM-F. Facta Universitatis Series Mech Eng..

[37] Haqbin A (2022). Comparing best-worst method and full consistency method in a fuzzy environment. Decis Sci Lett.

[38] Pamucar D, Deveci M, Canıtez F, Bozanic D (2020). A fuzzy Full Consistency Method-Dombi-Bonferroni model for prioritizing transportation demand management measures. Appl Soft Comput.

[39] Khan F, Ali Y (2021). A facilitating framework for a developing country to adopt smart waste management in the context of circular economy. Environ Sci Pollut Res.

[40] Stević Z (2020). Sustainable supplier selection in healthcare industries using a new MCDM method: Measurement of alternatives and ranking according to COmpromise solution (MARCOS). Comput Ind Eng.

[41] [NICE], N.I.f.H.a.C.E. 2013.

[42] Alkhamis AA (2017). Critical analysis and review of the literature on healthcare privatization and its association with access to medical care in Saudi Arabia. J Infect Public Health.

[43] Ravaghi, H.R., et al., *A holistic view on implementing hospital autonomy reforms in developing countries: a systematic review.* Health Policy and Planning, 2018: p. 1–10. 10.1093/heapol/czy095 30544175

[44] Bodner A (2022). Exploring privatization in Canadian primary care: an environmental scan of primary care clinics accepting private payment. Healthc Policy.

[45] Ravaghi H (2018). A holistic view on implementing hospital autonomy reforms in developing countries: a systematic review. Health Policy Plan.

[46] London JD (2013). The promises and perils of hospital autonomy: Reform by decree in Viet Nam. Soc Sci Med.

[47] Shen Y-C (2003). Changes in hospital performance after ownership conversions. Inquiry.

[48] Liu GG (2020). Does ownership matter for medical system performance? Evidence from a natural experiment in Suqian, China. Inquiry.

[49] Villa S, Kane N (2013). Assessing the impact of privatizing public hospitals in three American states: implications for universal health coverage. Value in Health.

[50] Sehngelia L, Pavlova M, Groot W. Impact of healthcare reform on universal coverage in Georgia: a systematic review. Divers Equal Health and Care, 2016.

[51] Collins D (1999). Hospital autonomy: the experience of Kenyatta National Hospital. Int J Health Plan Manag.

[52] Wagstaff A, Bales S. The impacts of public hospital autonomization: evidence from a quasi-natural experiment. Policy Research Working Paper, No. 6137. World Bank, Washington, DC, 2012.

[53] Maharani A, Femina D, Tampubolon G (2014). Decentralization in Indonesia: lessons from cost recovery rate of district hospitals. Health Policy Plan.

[54] Picone G, Chou S-Y, Sloan F (2002). Are for-profit hospital conversions harmful to patients and to Medicare?. RAND J Econ.

[55] Pirozek P (2015). Corporate governance in Czech hospitals after the transformation. Health Policy.

[56] Sharma S, Hotchkiss D (2001). Developing financial autonomy in public hospitals in India: Rajasthan's model. Health Policy.

[57] Kim T, McCue M (2012). The performance of the leveraged buyout of the Hospital Corporation of America Inc. Health Care Manag Rev..

[58] Pan J, Qin X, Hsieh CR (2016). Is the pro-competition policy an effective solution for China’s public hospital reform? Health economics. Health Econ Policy Law.

[59] Ramamonjiarivelo Z (2020). The privatization of public hospitals: its impact on financial performance. Med Care Res Rev.

[60] Buzelli ML, Boyce T (2021). The privatization of the italian national health system and its impact on health emergency preparedness and response: the COVID-19 case. Int J Health Serv.

[61] Tiemann O, Schreyögg J (2012). Changes in hospital efficiency after privatization. Health Care Manag Sci.

[62] Manheim L, Shortell S, McFall S (1989). The effect of investor-owned chain acquisitions on hospital expenses and staffing. Health Serv Res.

[63] Ramamonjiarivelo Z, Hearld L, Weech-Maldonado R (2021). The impact of public hospitals' privatization on nurse staffing. Health Care Manage Rev.

[64] Maharani A, Tampubolon G (2016). Does corporatisation improve organisational commitment? Evidence from public hospitals in Indonesia. Int J Hum Resour Manag.

[65] Niakas D, Mylonakis J (2005). Choice of physician, private payment and patient satisfaction. Is there any relationship?. Int J Healthc Technol Manag.

[66] Arndt M, Bigelow B (1996). Benefits and disadvantages of corporate restructuring–the hospital view. Hosp Top.

[67] Huang J, Shi L, Chen Y (2013). Staff retention after the privatization of township-village health centers: a case study from the Haimen City of East China. BMC Health Serv Res.

[68] Marathe S (2020). The impacts of corporatisation of corporatisation of healthcare on medical practice and professionals in Maharashtra, India. BMJ Glob Health.

[69] Fard ZR (2020). The association between nurses' moral distress and sleep quality and their influencing factor in private and public hospitals in Iran. J Educ Health Promot.

[70] Mohammadi M, Rezaei J (2020). Bayesian best-worst method: a probabilistic group decision making model. Omega.

[71] Jones GH, Kantarjian H (2015). Health care in the United States—basic human right or entitlement?. Ind Corner Perspect Controv.

[72] Maruthappu M, Ologunde R, Gunarajasingam A (2012). Is health care a right? Health reforms in the USA and their impact upon the concept of care. Ann Med Surg (Lond).

[73] Tiemann O, Schreyögg J, Busse R (2012). Hospital ownership and efficiency: a review of studies with particular focus on Germany. Health Policy.

[74] Lee SL, Yaghoubian A, Kaji A (2012). County versus private hospitals: access of care, management and outcomes for patients with appendicitis. JSLS.

[75] Pan J (2016). Assessing spatial access to public and private hospitals in Sichuan, China: the influence of the private sector on the healthcare geography in China. Soc Sci Med.

[76] Kruse FM (2018). Do private hospitals outperform public hospitals regarding efficiency, accessibility, and quality of care in the European Union? A literature review. Int J Health Plann Manage.

[77] Yildiz MS, Heboyan V, Khan MM (2018). Estimating technical efficiency of Turkish hospitals: implications for hospital reform initiatives. BMC Health Serv Res.

[78] Jing R (2019). Technical efficiency of public and private hospitals in Beijing, China: a comparative study. Int J Environ Res Public Health.

[79] Garmatz A, Vieira GBB, Sirena SA (2021). Assessing the technical efficiency of Brazil's teaching hospitals using data envelopment analysis. Cien Saude Colet.

[80] Preker AS, Harding A, Travis P (2000). "Make or buy" decisions in the production of health care goods and services: new insights from institutional economics and organizational theory. Bull World Health Organ.

[81] Preker AS, Harding A (2005). The economics of hospital reform from hierarchical to market-based incentives. World Hosp Health Serv.

[82] Preker AS, Langenbrunner JC (2005). The role of purchasing in hospital performance. World Hosp Health Serv.

[83] Sobhani S (2019). From privatization to health system strengthening: how different International Monetary Fund (IMF) and World Bank policies impact health in developing countries. J Egypt Public Health Assoc.

[84] Tabrizi JS, Aghdash SA, Nouri M (2021). Countries' experiences in reforming hospital administration structure based on the Parker and Harding model: a systematic review study. J Educ Health Promot.

[85] Maharani A, Tampubolon G (2017). The double-edged sword of corporatisation in the hospital sector: evidence from Indonesia. Health Econ Policy Law.

[86] Waitzkin H, Jasso-Aguilar R, Iriart C (2007). Privatization of health services in less developed countries: an empirical response to the proposals of the World Bank and Wharton School. Int J Health Serv.

[87] Oliver T, Schreyögg J (2012). Changes in hospital efficiency after privatization. Health Care Manag Sci.

[88] Doshmangir L (2015). Opening the black box: the experiences and lessons from the public hospitals autonomy policy in Iran. Arch Iran Med.

[89] Dahlgren G (2014). Why public health services? Experiences from profit-driven health care reforms in Sweden. Int J Health Serv.

[90] Sohrabi R (2021). A scoping review of public hospitals autonomy in Iran: from budgetary hospitals to corporate hospitals. BMC Health Serv Res.

